# Hearing impairment and vestibular function in patients with a pathogenic splice variant in the *LHX3* gene

**DOI:** 10.1186/s12920-024-02049-5

**Published:** 2024-11-15

**Authors:** Åsa Kjellgren, Elenor Lundgren, Irina Golovleva, Berit Kriström, Mimmi Werner

**Affiliations:** 1 Department of Clinical Sciences, Otorhinolaryngology, University of Umeå, Umeå, Sweden; 2 Department of Medical Biosciences, Medical and Clinical Genetics, University of Umeå, Umeå, Sweden; 3 Department of Clinical Sciences, Pediatrics, University of Umeå, Umeå, Sweden

**Keywords:** Child, Founder mutation, Pituitary hormone deficiency, CPHD, Sensorineural hearing loss, Balance impairment, Progressive hearing loss

## Abstract

**Background:**

*LHX3* is a gene encoding a LIM-homeodomain transcription factor important for the fetal development of several organs, such as the pituitary gland, spinal motor neurons and the inner ear. Pathogenic and likely pathogenic variants in the *LHX3* gene are infrequent and result in a rare syndrome known as combined pituitary hormone deficiency-3, CPHD3.

**Methods:**

We have studied hearing and vestibular functions in a group of eight individuals, aged 8–36 years, all of whom were homozygous for a specific variant in the *LHX3* gene at chromosome 9q34. We reexamined the results of consecutive hearing tests from newborn until April 2024.

**Results:**

Our data showed that all the tested patients had progressive sensorineural hearing deficiency ranging from moderately severe to complete loss. We have performed vestibular testing in six patients and, for the first time, demonstrated that a mutation in the *LHX3* gene not only affects hearing, but is also associated with vestibular impairment.

**Conclusion:**

The human pathogenic variant c.455-2A > G in the *LHX3* gene on chromosome 9q34, which present as a founder mutation in the population in northern Sweden, is responsible for phenotypes associated with progressive hearing loss and balance impairment. These findings prove that the *LHX3* gene is crucial for the function of both the cochlear and vestibular organs.

## Introduction

In northern Sweden, we have identified a cohort of eight patients, all of whom had clinical presentation characteristics of a rare syndrome known as combined pituitary hormone deficiency 3 (CPHD3; OMIM 221750) with combined pituitary hormone deficiency (CPHD), a short neck with restricted movement and vertebral malformations. Six of the patients in this cohort were previously described to have CPHD3 combined with sensorineural hearing loss [12]. Molecular genetic analysis revealed that all patients were homozygous for the same variant in the *LHX3* gene at chromosome 9q34. The *LHX3* c.455-2A > G (NM_178138.6) variant is predicted to abolish the splice acceptor site in intron 3, which would result in a truncated protein lacking both the homeodomain and the carboxyl terminus of the normal LHX3 protein [12]. It is also classified as pathogenic according to ACMG guidelines [20]. Currently, only 32 *LHX3* variants are reported in The Human Gene Mutation Database (HGMD)® Professional 2023.4 (http://www.hgmd.cf.ac.uk/ accessed on 14th of March 2024), representing missense, nonsense and splicing mutations,one gross deletion,and one small indel. Three studies reported that fewer than 10% (3 out of 32) of the variants were splicing variants [1, 12, 24]. All *LHX3* variants were annotated as disease mutations associated with pituitary hormone deficiency. There are several published case-reports describing patients with similar phenotypes associated with biallelic *LHX3*, although all the *LHX3* variants seem to be novel. To the best of our knowledge, no other clusters of individuals with this syndrome caused by a founder mutation have been reported [1–4, 12, 14, 16–19, 24]. Hearing impairment of varying severity has been reported for some but not all CPHD3 patients [1, 2, 4, 12, 14, 18, 19, 24]. Recently, monoallelic *LHX3* mutation carriers with a milder phenotype and limited neck rotation or milder CPHD have been described. None of these symptomatic heterozygotes were reported to have hearing impairment [8, 24]. To the best of our knowledge, there are no reports regarding vestibular function in individuals with mutations in the *LHX3* gene.

The *LHX3* gene encodes a LIM-homeodomain transcription factor that is expressed early in fetal life and is important for the development of several different organs. This transcription factor is a key regulator of pituitary development [10, 22, 23]. *Lhx3-* null mice die at birth, and studies of embryos from *Lhx3-* null mice have shown an increased apoptosis in the pituitary gland [5]. In mammals, the *LHX3* gene is required for the development of spinal motor neurons [21]. Dogs with mutations in the *LHX3* gene have CPHD and vertebral malformations [27]. In mice, the *Lhx3* gene is expressed exclusively in the hair cells of both the auditory and the vestibular systems [6, 7]. In vestibular organs, this expression is maintained until adulthood, while in the cochlea, Lhx3 is downregulated after birth [7]. The development of the inner ear is dependent on a cascade of different transcription factors from the LHX, SOX and basic helix-loop-helix (bHLH) families [7, 29]. The role of LHX3 in this cascade is only partly understood.

In human embryos, *LHX3* is expressed in the vestibular epithelium during early fetal development, from around day 49 and forward [18]. In contrast, *LHX3* was not found in the cochlear epithelium, and it was suggested that *LHX3* expression in the cochlea might evolve later since the hair cells in the cochlea develop later than those in the vestibular organs. They studied human fetal material for up to 9 weeks of development, and the auditory hair cell differentiation has previously been shown to start at 11–12 weeks [13, 18].

SRY-box 2 (SOX2) is another transcription factor that has been found to be important for the development of human cochlear and vestibular organs [11]. The areas where *Lhx3* is expressed overlap the areas where the transcription factor *Sox2* is expressed during the development of the inner ear in mice [7].

The same overlapping pattern between *LHX3* and *SOX2* has also been reported in human embryos [18]. In both mouse and human vestibular organs and in the mouse cochlea, the expression of *SOX2* precedes the expression of *LHX3* [7, 18], and SOX2 can activate LHX3 expression in vitro [18]. The *Lhx3* gene is regulated by the transcription factor Pou4f3 in the auditory, but not in the vestibular, hair cells of mice [6]. In humans, a dominant mutation of the gene encoding the Pou4f3 transcription factor is responsible for the nonsyndromic progressive hearing loss DFNA15 [25].

The aim of this study was to evaluate the impact of the *LHX3* c.455-2A > G variant on hearing and balance in eight CPHD3 patients. Specifically, we intended to study hearing impairment and its progression in a long-term follow-up setting in a cohort of biallelic *LHX3* syndrome patients and to investigate whether a vestibular impairment is associated with this mutation.

## Subjects and methods

### Ethics approval

This study was performed in accordance with the principles of the Declaration of Helsinki. Approval was granted by the National Ethics Committee of Sweden (2023–02-10/2022–06806-01).

### Consent to participate

Informed consent was obtained from all individual adult participants and from all children´s parents included in the study.

### Subjects

Eight patients, seven males and one female, currently 9–38 years of age were included in our study. All eight patients had CPHD with a deficiency in growth hormone, thyroid-stimulating hormone, prolactin, lutenizing hormone and follicle-stimulating hormone and typical stigmata as described by Kriström et al. [12]. Hormone supplementation therapy for CPHD started during the neonatal period.

**Fig. 1:**
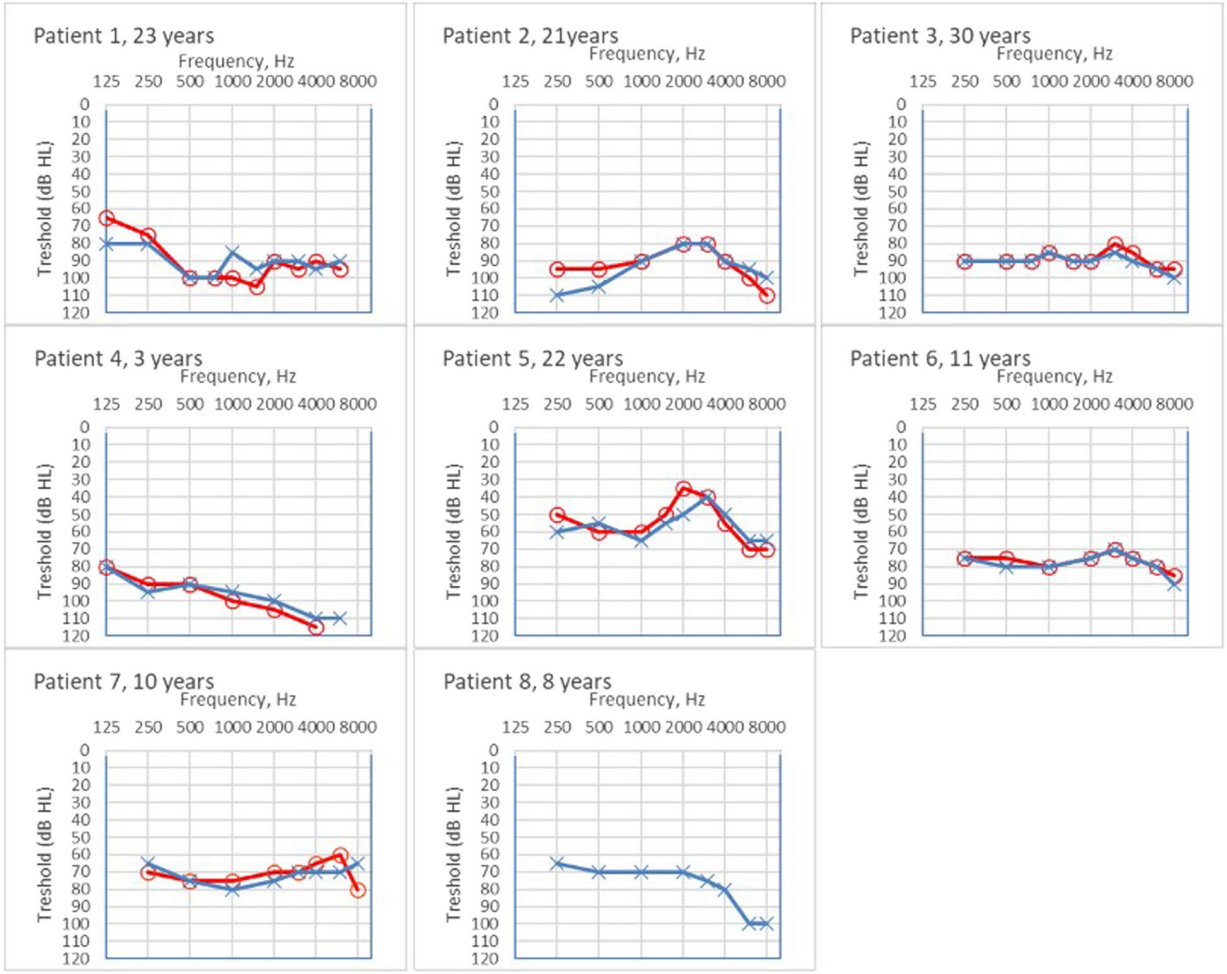
Individual hearing tests today. Figure shows the latest hearing tests. For patients 1, 3 and 6, the last hearing test before cochlear implantation is shown. For patient 8, only the nonimplanted ear was included

One of our patients died at the age of 17 due to complications from a vertebral surgery for his scoliosis. Six of our patients speak spoken Swedish and two have sign language as their first language. (Table 1). All eight patients were born in Västerbotten or nearby counties and all of them had undergone several hearing tests since early childhood and onward and had been diagnosed with various degrees of hearing loss. (Table 1 and Fig. 1).

**Table 1:** Hearing impairment

Patient	Year of birth (age)	Gender	Developmental or neuro-psychiatric diagnosis	Age at diagnosis for hearing loss	Hearing loss at diagnosis	Hearing loss at latest hearing test	PTA4 best ear^b^	WRS with HA or CI	Language	Means of hearing
1	1985 (38)	m		7 months	Moderately severe or severe	Complete	96 dB	84% (Bilateral CI)	Swedish	CI + CI
2	1990 (33)	f	Developmental disorder	9 months	Severe	Profound	89 dB		Sign language	none
3	1992(31)	m		19 months	Moderately severe	Profound	88 dB	70% (bilateral HA)	Swedish	HA
4	1995^a^	m	Developmental disorder	4 months	Profound	Complete	99 dB		Sign language	none
5	2000(23)	m		5 years	Mild	Moderately severe	53 dB		Swedish	HA
6	2007(16)	m	Autism level 1	15 months	Uncertain, moderate to severe	Severe	70 dB	72% (left CI)	Swedish	CI + HA
7	2013(10)	m		6 months	Moderate	Moderately severe	60 dB	86% (bilateral HA)	Swedish	HA
8	2014(9)	m	Autism level 2	2 months	Moderately severe	Severe	76 dB	80% (right CI)	Swedish	CI + CI

*CI* Cochlear Implant, *HA* Hearing Aid

^a^Deceased at age 17, ^b^Latest hearing test or last hearing test before CI

In addition to the eight patients included in this study we have also found a 9th patient with the syndrome. This patient was born in 1988, had a brain hemorrhage in early life and suffered from epilepsy and severe developmental disorder. He died at 8 years of age from epileptic seizures. This child was posthumously diagnosed with CPHD3 based on typical stigmata judged by investigators from family photos combined with the molecular-genetic testing results of parental DNA that showed both parents being carriers of pathogenic *LHX3* variant.

### Molecular-genetic testing of *LHX3* and genealogical studies

Sanger sequencing was applied for detection of *LHX3* variant c.455-2A > G (NM_178138.6). Amplification of the fragment covering intron–exon junction of exon 4 and partial sequence of exon 4 was performed on genomic DNA. Primers for *LHX3* amplification designed with Primer3 software are following, forward – 5’- tgtaaaacgacggccagt CCTTCCGAGAAGCCTGTG and reverse – 5’- caggaaacagctatgacc CATGTCCAGGCCCGTCTC. Both primers have been tagged with M13- sequence. PCR amplification was performed in 25 µl reactions with 5 U of KAPA2G Robust HS DNA Polymerase (Roche, IN, USA), 5 µl of PCR buffer 5xKAPA2G GC (Roche), 0.5 µl of 10 mM dNTP mix, 2 µl of primers mix, 1 µl DMSO and 50 ng of genomic DNA. PCR cycling consisted of an initial denaturing step at 95ºC for 5 min; then 40 cycles of denaturing at 95ºC for 15 s, annealing at temperature 61ºC) for for 15 s, and extension at 72ºC for 30 s; and a final elongation step at 72ºC for 1 min. PCR products were purified using MicroSpin columns (Amersham Biosciences, Piscataway, NJ, USA). The sequencing reactions were performed using Big Dyes Terminator v3.1 Cycle Sequencing Kit (Applied Biosystems, Foster City, CA, USA) in a final reaction of 10 ml. The products of sequencing reactions were run on a 3730xl DNA analyzer (Applied Biosystems). Sequences were aligned and evaluated using the Sequencher software version 4.9 (Gene Codes Corporation, Ann Arbor, MI, USA).

**Fig. 2:**
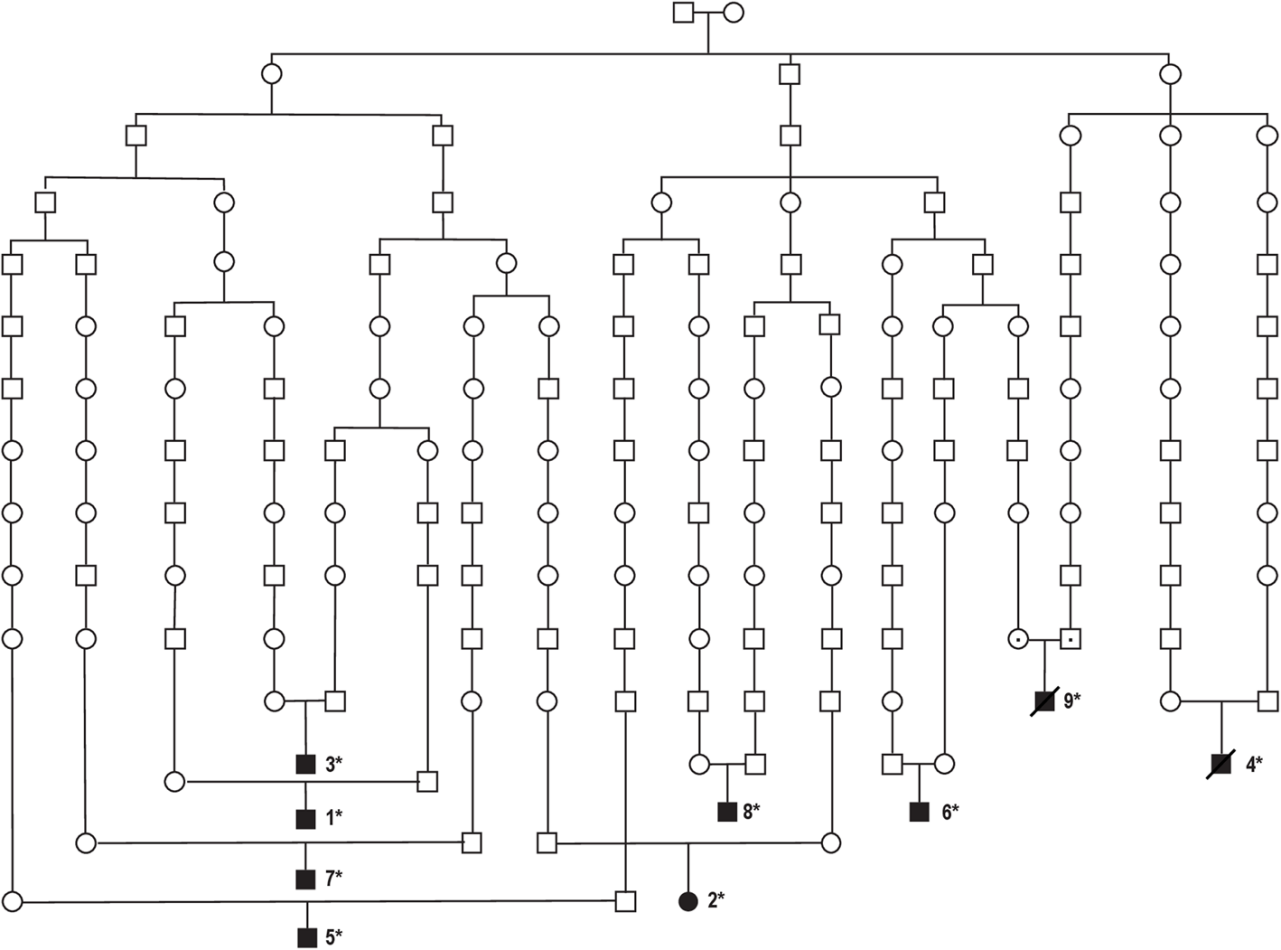
An extended pedigree showing nine individuals with recessive CPHD3 originating from northern Sweden. Patients 1 to 8 were homozygous for the *LHX3* c.455-2A > G variant. Patient 9 was diagnosed based on the results of parental testing that showed both parents being heterozygotes for *LHX3* c.455-2A > G variant (shown with a dot on the respective symbols) and typical CPHD3 stigmata. According to the results of the genealogical studies, all nine patients can be traced to the same ancestor 10–13 generations ago. Affected individuals are shown in shaded black and healthy subjects are shown as open circles for females and squares for males. Patients 4 and 9 are diseased. The individuals for whom DNA was available for this study are marked with asterisk. Initially, the pedigree with six cases was reported by Kristrom et al*.* [12],new family members have been added to the present version and the pedigree was modified

Genealogical studies revealed that all eight patients could be traced to the same ancestor in the seventeenth century (Fig. 2). Genealogical data also showed that the diseased patient 9 was related to the patients included in the study (Fig. 2).

### Hearing

#### Historical audiological data

To evaluate the development of the hearing impairment in each patient, the patients’ medical records were revisited, and all hearing test data from newborn until today were gathered. If a hearing test of a child was marked as very insecure or if the child had middle ear effusion according to the journal, that hearing test was excluded. If there were many hearing tests with similar results for one person within one year, only the first hearing test from that year was included. Only tests that were executed at an audiology department by specialized audiologists with calibrated equipment used in clinical routine at the time of testing were gathered. The equipment and protocol may have varied over the years.

#### Hearing tests

Hearing tests were evaluated for each patient, including otoscopic examination, tympanogram, and audiometry, with methods appropriate for the patient’s age. All hearing tests were conducted at an audiology department by specialized audiologists with calibrated equipment used in clinical routine. The methods for visual reinforcement audiometry, play audiometry, pure tone audiometry and speech audiometry are described in the Swedish Audiological Methodbook, (SAME) [15].

#### Visual reinforcement audiometry (VRA)

The test was performed in a soundproof room. The child sat in the lap of the parent, and sound was presented in speakers on the right and left sides of the child. When the child looked for the sound, a dark Plexiglas box was lit, and an animated toy was shown to the child.

#### Play audiometry

Pure tone audiometry with play was performed in a soundproof room using headphones. The child was instructed to perform a simple task, such as moving a ball into a bucket or placing a brick on a tower every time they heard a sound.

#### Pure tone audiometry and bone conducting thresholds

Pure tone audiometry was conducted in a soundproof room using headphones. Bone conducting thresholds were measured in the same room using a bone conducting oscillator over the mastoid bone. Pure tone audiometry and bone conducting thresholds were both assessed according to the ISO standard 8253–1.

#### Speech audiometry with hearing aids

Speech audiometry was performed without background noise and in a soundproof room. The test was performed in sound-field and with the patient using their normal hearing aids or cochlear implants. A loudspeaker was placed one meter in front of the patient. A disc with a list of 50 monosyllabic words in the patient’s native language (Swedish) was played at a fixed level of 65 dB SPL. The word recognition score (WRS) was calculated as the percentage of correctly repeated words. For the children, a list of 25 words adapted according to the child’s age was used.

#### Auditory steady-state response (ASSR)

All ASSRs included in the study was performed with Interacoustics eclipse using a standard clinical protocol. The ASSR thresholds are estimated hearing level (dB eHL).

#### Auditory brainstem responses (ABR)

All ABRs included were performed using broadband stimuli, clicks or chirps. The hearing threshold was determined as the lowest level where a repeatable ABR curve was detectable. All ABR thresholds are in dB nHL.

#### Otoacoustic emissions (OAE)

Transient evoked otoacoustic emissions (TEOAE) has been used as a diagnostic tool in our department since the early 1990s and is the OAE method used in this study. Neonatal screening with otoacoustic emissions became routine for all children in Sweden in 2005. In this study, only OAEs taken at an audiological department by a specialized audiologist were included. The stimulus level varies between 35 dB in the 90th to 30 dB today, and the criterion for pass is responses at three of four frequencies, 1500 Hz—4000 Hz.

The 4-frequency pure- tone- average (PTA4) was calculated as the average of the hearing levels at 0.5, 1.0, 2.0 and 4.0 kHz. In VRA, sometimes the audiologist has been able to measure only two or three of these four frequencies. In that case, an average was calculated for the frequencies measured.

The severity of hearing loss was classified according to the WHO into five levels: mild (20–34 dB HL), moderate (35–49 dB HL), moderately severe (50–64 dB HL) severe (65–79 dB HL), or profound (> 80 dB HL).

### Balance

#### Posturomotor control milestones

Pediatric medical records were checked for posturomotor control milestones. When possible, the parents were also interviewed about the child’s early development.

### Vestibular testing

The patient’s vestibular function was investigated in our clinical setup which included the following:

#### Vestibular head impulse test (vHIT)

The vestibular head impulse test was performed with Interacoustics EyeSeeCam equipment. vHIT was considered pathological when the gain at 60 ms calculated from at least seven head movements was lower than 0.79 and when refixation saccades could be identified.

#### Caloric test

The caloric test was performed with the patient in supine position and the head in a position at 30 degrees elevation. Water was irrigated into the ear canal for 30 s in two set-ups; with cold (C), 30° C; respective warm (W), 44 °C; and water for the right (R) and left (L) ears. There was a pause between the irrigations, so the next irrigation was performed well after the nystagmus from the previous irrigation had calmed down. Eye movements were tracked, and the maximum velocity of the slow- phase component of the nystagmus was analyzed. A total response (CR + CL + WR + WL) of less than 20°/s was considered as bilateral hypofunction.

#### Cervical vestibular- evoked myogenic potentials (cVEMPs)

cVEMP was recorded with an Interacoustics eclipse machine using mastoid bone stimulation with a 500 Hz tone burst at 60 and 65 dB nHL. An electrode was placed over the SCM-muscle and the EMG responses were recorded. At least 200 responses were processed by filtering, averaging and amplification. A reproductive positive peak at 11–17 ms after stimulation followed by a negative peak at 19–25 ms was considered to indicate a response.

For the children who received cochlear implants at the Karolinska Hospital in Stockholm, the vestibular investigation prior to implantation was performed at Karolinska, and the vestibular assessment was performed for the children described in their validation study [26].

### Radiological examinations

MRI scans of the inner ear had been obtained for clinical reasons in five patients using 1.5 or 3 T and a standard clinical protocol for the inner ear with 0.6 -1 mm slices over the temporal bone.

## Results

### Molecular-genetic testing

All eight patients were homozygous for the c.455-2A > G variant in the *LHX3* gene (NM_178138.6) on chromosome 9q34. This variant was interpreted as pathogenic in accordance with ACMG guidelines [20].

### Hearing

All hearing tests available from newborn until today were evaluated. All eight patients have developed a sensorineural hearing loss ranging from moderately severe (patients 5 and 7) to severe (patients 6 and 8) or profound to complete (patients 1, 2, 3 and 4). (Table 1). The hearing loss for all patients was symmetrical between the ears and relatively flat, affecting all the frequencies (Fig. 1). Conducting thresholds confirmed that the hearing loss was sensorineural for all eight patients.

### Otological examination

All patients have had their ears otomicroscopically examined by an otorhinolaryngologist and no abnormalities were found.

### Tympanometry

Patients 5–8, as examined via tympanometry, had type A curves within the normal range.

### Hearing during the first year of life

Seven of our eight patients (patients 1, 2, 3, 4, 6, 7 and 8) had some form of hearing test results indicating the possibility of a hearing loss during their first year of life. The method of performing the first hearing test varied from wake-up audiometry in the 1980s and early 1990s to OAE, ABR and ASSR tests in the 2000s and 2010s. The age at diagnosis in these 7 patients ranged from two to 16 months, and the hearing levels at diagnosis varied from moderate to profound hearing loss. One patient (patient 5) had normal OAE at the age of two and was diagnosed with mild hearing loss at the age of five (Table 1).

### OAE

Four patients (patients 3, 4, 6 and 8) examined with TEOAE during their first year (aged 12, 2, 7 and 1 month, respectively) did not pass the test. TEOAE was normal for patient 5 examined at 2 years of age. This patient returned after preschool screening at the age of four and still had a normal TEOAE, but his play audiometry indicated a mild hearing loss (Table 1).

### Progression of hearing loss

**Fig. 3:**
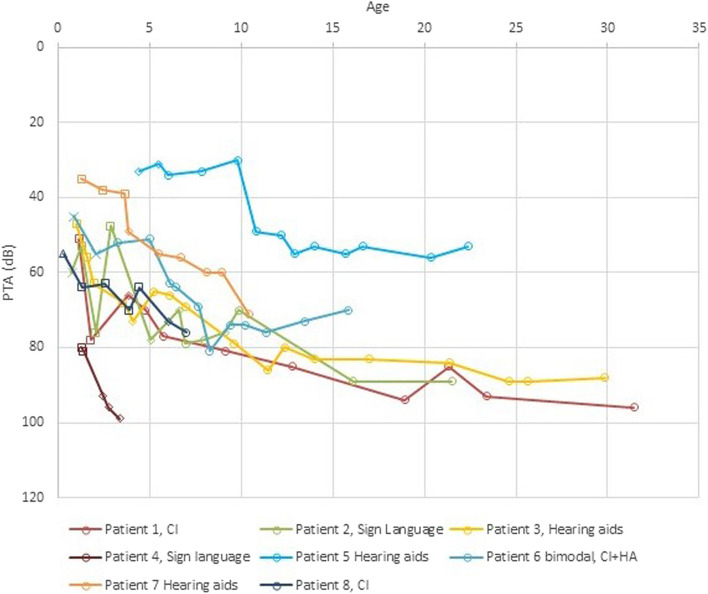
Changes in average hearing thresholds over time in the best ear. For ASSR, VRA, play audiometry and pure tone audiometry the figure shows the average of the hearing levels at 0.5, 1.0, 2.0 and 4.0 kHz. For BRA the figure shows the minimum level of a broadband stimuli needed to get a response. Method used to measure hearing: ∆ ASSR x BRA □ Visual Reinforcement audiometry ◊ Play audiometry ○ Pure tone audiometry

All eight patients had a progressive hearing loss (Fig. 3). The loss in the PTA in the best ear from the first reliable hearing test to the present day ranged from 19 to 45 dB with a mean progression of 29 dB. Regarding the currently adults, most of the hearing level progression occurred during their first 12 years of life, but a slow progression has continued throughout their life.

### Cochlear implants (*CIs*)

None of our patients received cochlear implants as infants. Patient 4 was born in 1995 with profound hearing loss and received a cochlear implant at 3 years of age but became a nonuser. Patients 1, 6 and 8 were born with moderate or moderately severe hearing loss and have all received cochlear implants later due to progression of the hearing loss. The patients received their first cochlear implant at ages 23, 12 and 4 years respectively. Patients 1 and 8 wear bilateral cochlear implants today, and patient 6 is bimodal with a cochlear implant on his left ear and a hearing aid on his right. These three patients all had good results according to speech audiometry, with a WRS of 72–84% with their implants in sound field (Table 1).

### Balance

#### Posturomotor control milestones

The age at which the patients walked independently varied from 16 to 24 months, with a mean walking age of 19.5 months. This is clearly above the mean walking age of Swedish children with no balance impairment and in parity with the mean walking age of children with congenital CMV infection with documented balance impairment [9]. Almost all children in Sweden learn to ride a bicycle. In northern Sweden skiing and skating are also mandatory in school. When asked, most of our patients reported either being substantially later than their peers in learning to ride a bicycle (2 patients) or not at all being able to ride a two-wheel bicycle (4 patients). They also reported problems learning other types of balance requiring sports, such as skating or skiing. Some of them had extra training at school or attended a physiotherapist to improve balance. Several problems with the balance were reported by six of eight patients or their parents (Table 2).

**Table 2:** Balance

Patient	Age	Walking age (months)	Biking	Problems with balance	Caloric irrigation	vHIT	VEMP
1	38	18	No	Yes	No responses	hypofunction	No responses
2	33	20	No	Yes	-	-	-
3	31	18	Yes (late)	Yes	No responses	hypofunction	No responses
4	*	18	Yes	No	-	-	-
5	23	16	Yes(late)	Yes	No responses	Restricted neck movement	No responses
6	16	24	Yes	Small	No responses	hypofunction on left side	Asymmetrical Responses on right side but not on left side
7	10	21	No	Yes	-	Normal	-
8	9	21	No	No	No responses	normal	Normal

^*^Deceased at age 17

### Vestibular testing

Vestibular function was assessed in six of our patients, three adults (patients 1, 3 and 5) and three children (patients 6, 7 and 8), using caloric irrigation, vHIT and cVEMP. Patient 7 was tested with only the vHIT (Table 2). None of the patients tested had a normal caloric reaction. The three adults were tested with both warm and cold water, while two children (patients 6 and 8) were tested with cold water (30 °C) only due to difficulties in participation.

vHIT was tested in six patients. One of the adults (patient 5) had very restricted neck movements, making it impossible to obtain a reliable test. Of the remaining five patients, the two adults (patients 1 and 3) had pathological vHIT on both sides in all three directions. One of the three children (patient 6) had a pathological vHIT on the side with the cochlear implant, and the other two children (patients 7 and 8) had normal reactions on both sides.

cVEMP was tested in five patients. The three adults (patients 1, 3 and 5) had no response. Of the two children, one (patient 8) had normal responses in both ears and the other (patient 6) had responses in the ear without cochlear implant.

The balance of two of the children was also evaluated prior to cochlear implantation. One patient (patient 8) with vHIT and VEMP and the other one (patient 6) with caloric “ice water” irrigation and VEMP, and both had normal results on these tests.

### MRI

MR images of the temporal bone were obtained for five of our patients (patients 1, 3, 5, 6 and 8). All five had normal anatomy of the inner ear and acoustic nerve channel. Patient 5 had a 1 mm segment in the ductus cochlearis with a reduced signal. In the other four patients, we found no abnormalities.

### Neuro-developmental disorders

Neurodevelopmental disorders were not the focus of this study but during the data collection we found that two of our eight patients were diagnosed with developmental disorder and two others with autism.

## Discussion

In this study we show for the first time that a pathogenic variant in the *LHX3* gene affects the function of both the cochlear and vestibular organs. The *LHX3* gene is expressed in the hair cells of both cochlear and vestibular organs, but there are no previous reports on the balance of patients with mutations in the *LHX3* gene. We also report that the hearing impairment progresses over time for the patients included in this study. These clinical findings are important for obtaining a better understanding of the role of LHX3 in the development of the inner ear and will also have direct clinical implications for the treatment of the patients with CPHD3.

Our clinical findings indicate that LHX3 is an important transcription factor involved in the development of both cochlea and vestibular organs. These findings are consistent with previous findings in mice showing that Lhx3 is expressed in both cochlear and vestibular hair cells during periods critical for inner ear development [7].

Most of our patients reported various degrees of balance problems. They have been late walkers with a walking age similar to what is seen in other conditions affecting vestibular function, such as congenital CMV [9]. Although it can be argued that other symptoms in the CPHD3 syndrome, such as restricted neck movement and spinal problems, may also affect motor skills and walking age, our vestibular testing indeed showed that there was a loss of vestibular function associated with this syndrome.

Bilateral vestibular loss is quite uncommon in the population, with a prevalence of 28/100 000 adults [28]. Our three adult patients demonstrated a total loss of vestibular function in caloric tests, vHIT and VEMP. One of these patients received bilateral cochlear implants in adulthood wich can affect balance, but he had balance problems since childhood indicating that the balance impairment might have been present earlier. Unfortunately, we have no balance tests prior to the cochlear implants for this patient. For the other two adults, we have no explanation other than that vestibular loss is part of the syndrome.

Our results from balance tests differ between adults and children. The three adults had no balance function in any of our tests while the three children had responses in the vHIT and in the two tested also in VEMP but not in caloric testing. One possible explanation is that the balance impairment is progressive, which is in line with the progressive pattern of hearing impairment that we showed in this study.

One child had a lack of vestibular function in the left ear where he has a cochlear implant but normal vHIT and VEMP in the nonimplanted ear. Prior to implantation he had normal VEMP on both sides. In this case the lack of vestibular function in the implanted ear was probably induced by the cochlear implantation.

Our findings showed that the hearing loss in our patients with CPHD3 syndrome was progressive, especially during childhood, and that children born with moderate or moderately severe hearing loss may progress to severe or profound forms. Therefore, follow- up is important for adjusting hearing-aids when needed. Furthermore, for this group cochlear implants are a very good alternative when normal hearing-aids are not sufficient.

Hearing levels vary greatly between individuals with this syndrome. Six out of eight of our patients currently have severe or profound hearing loss. Looking back on the hearing of our patients at an early age, only two of the eight patients had severe or profound hearing loss when their hearing loss was diagnosed.

We reviewed case reports on CPHD3 syndrome and found a total of 14 patients whose hearing was examined. Ten of these patients were reported to have mild or moderate hearing loss [1, 4, 18, 19], only three had severe or profound hearing loss [18, 24] and one patient had normal hearing [14].

The patients in this study had a more severe hearing impairment than did those in previous case-reports. However, our patients were older, and their hearing impairment progressed over time. Four of our eight patients were adults, however, in the case studies mentioned above, at least half of the patients were children under five years old at the time of the hearing test, and only one out of 14 patients were adults [1, 4, 14, 16, 18, 19, 24].

We have not found any follow- up reports regarding hearing for these previously published cases, but it would be very interesting to know if their hearing impairment is also progressive.

In addition to the eight patients included in the study we identified a 9th patient with the syndrome. Due to the lack of hearing- or balance-tests this patient was excluded from the study. The fact that all the other patients in our cohort had hearing impairment led us to suspect that he might also have had hearing loss, but at the time his genetic variant was not known and he had no suspected hearing disability; therefore, his hearing was never properly tested. This case shows the importance of testing the hearing in all children, especially those who have other disabilities.

The sex distribution in our group was uneven, with seven males and one female. Case reports from other countries have shown a more even sex distribution, with 14 boys and 11 girls in 10 different studies [1–4, 14, 16–19, 24]. These findings suggested that the large number of boys in our cohort could be a mere coincidence.

Two out of the eight patients included in this study were diagnosed with developmental disorder and two with autism. The ninth patient, excluded from the study, also had developmental disorder. Neurodevelopmental disorders were not the focus of this study, but these findings raises the question if these disorders might also be a part of the syndrome.There are other case-reports mentioning the child having mental retardation. [1, 3, 17, 18].

The strengths of this study are the large number of patients and the long-term follow-up on the hearing tests proving a previously unknown progression of the hearing loss and, also the vestibular findings showing for the first time that balance impairment is associated with the syndrome. A weakness is that the retrospective setup of the study inevitably entails lack of some data as vestibular data from the early years of the today adult patients.

## Conclusions

The human pathogenic variant c.455-2A > G in the *LHX3* gene on chromosome 9q34, which is a founder mutation in the population in northern Sweden, is responsible for phenotypes associated with progressive hearing loss and balance impairment, in addition to CPHD and vertebral malformations. These findings prove that the *LHX3* gene is crucial for the function of both the cochlear and vestibular organs.

Children with CPHD3 should have their hearing and balance assessed early in childhood. Due to the progressive nature of the impairment, continuous follow-ups are essential.

## Data Availability

Data is provided within the manuscript.
