# Correction: Hearing impairment and vestibular function in patients with a pathogenic splice variant in the *LHX3* gene

**DOI:** 10.1186/s12920-024-02062-8

**Published:** 2024-12-09

**Authors:** Åsa Kjellgren, Elenor Lundgren, Irina Golovleva, Berit Kriström, Mimmi Werner

**Affiliations:** 1 Department of Clinical Sciences, Otorhinolaryngology, University of Umeå, Umeå, Sweden; 2 Department of Medical Biosciences, Medical and Clinical Genetics, University of Umeå, Umeå, Sweden; 3 Department of Clinical Sciences, Pediatrics, University of Umeå, Umeå, Sweden

**Correction to: Kjellgren** ***et al. BMC Medical Genomics (2024) 17:270***

10.1186/s12920-024-02049-5

Following the publication of the original article, the authors reported the correct LHX3 variant is c.455–2 A > G (**NM_178138.6**), instead of NM_014564.5. In the PDF file, the wrong transcript is written on page 2 (Introduction), page 4 (Molecular-genetic testing of LHX3 and genealogical studies) and page 7 (Results, Molecular-genetic testing).

The Original Article has been corrected.

